# Systems and WBANs for Controlling Obesity

**DOI:** 10.1155/2018/1564748

**Published:** 2018-02-01

**Authors:** Maali Said Mohammed, Sandra Sendra, Jaime Lloret, Ignacio Bosch

**Affiliations:** ^1^University of Gezira, Wad Madani, Sudan; ^2^Universitat Politècnica de València, Valencia, Spain; ^3^Departamento de Teoría de la Señal, Telemática y Comunicaciones, Universidad de Granada, Granada, Spain

## Abstract

According to World Health Organization (WHO) estimations, one out of five adults worldwide will be obese by 2025. Worldwide obesity has doubled since 1980. In fact, more than 1.9 billion adults (39%) of 18 years and older were overweight and over 600 million (13%) of these were obese in 2014. 42 million children under the age of five were overweight or obese in 2014. Obesity is a top public health problem due to its associated morbidity and mortality. This paper reviews the main techniques to measure the level of obesity and body fat percentage, and explains the complications that can carry to the individual's quality of life, longevity and the significant cost of healthcare systems. Researchers and developers are adapting the existing technology, as intelligent phones or some wearable gadgets to be used for controlling obesity. They include the promoting of healthy eating culture and adopting the physical activity lifestyle. The paper also shows a comprehensive study of the most used mobile applications and Wireless Body Area Networks focused on controlling the obesity and overweight. Finally, this paper proposes an intelligent architecture that takes into account both, physiological and cognitive aspects to reduce the degree of obesity and overweight.

## 1. Introduction

Sedentary jobs, technology advancements, and unhealthy foods are the key factors that lead to global obesity prevalence. The global obesity rate is dramatically increasing. This problem has become a challenge facing public healthcare systems and global economies [1].

The main cause of obesity and overweight is an energy imbalance between consumed and expended calories because of the current lifestyle where the new forms of work are more linked to computers and the long working days are causing an increase in the sedentary behavior and the reduction of the physical activity (PA) of the population. This situation also encourages the ingestion of high-calorie intake food and fast food.

**Figure 1:**
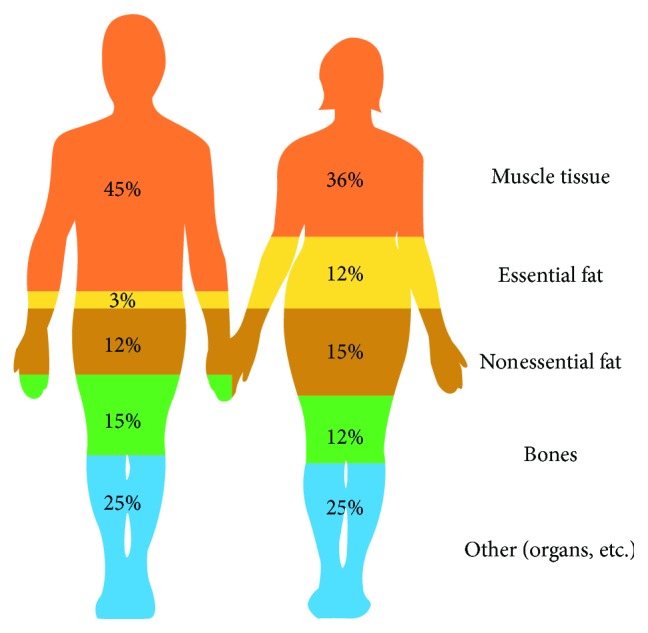
Healthy body composition.

As an indication, Figure 1 shows the composition of a healthy body for men and women [2]. In general, changes in eating habits and PA are the results of environmental and social changes associated with the social development and the lack of supportive policies in sectors such as health, the agriculture, transport, urban planning, environment, processing, distribution, and marketing of food and education.

Overweight and obesity are considered as one of the major risk factors for noncommunicable diseases such as cardiovascular diseases that were, in 2012, the leading cause of death, diabetes, musculoskeletal disorders, and some cancers [3]. The risk for these noncommunicable diseases increases when the body mass index (BMI) increases. Moreover, childhood obesity is associated with an increased likelihood of obesity, premature death, and disability in adulthood. In addition to these future risks, obese children experience breathing difficulties, bigger risk of fractures, and hypertension and have early markers of cardiovascular disease, insulin resistance, and psychological effects.

Overweight and obesity as well as noncommunicable diseases related to overweight are preventable. In this sense, people can choose to limit the energy intake of total fat and sugars and increase the consumption of fruits and vegetables as well as legumes and cereals and perform regular PA. Individual responsibility can only be fully effective if people have access to a healthy lifestyle. Moreover, at the social level, it is important to help people to follow those recommendations through the sustained implementation of demographic policies based on scientific evidence which would allow regular PA and healthier food options available and affordable to all, particularly for the poorest people. Countries should exert great efforts to solve this problem with an emphasis on promoting healthy eating culture and adopting the physical activity lifestyle. Advancement in modern technology has contributed to reducing the dilemma of obesity, which leads to many health complications by providing new, widely used, lightweight, easy to use, and low-cost consumer-based fitness wearable trackers and food intake systems [4]. These helpful tools provide an objective indicator of a user's daily energy expenditure (EE), PA, and food history over long periods. Users can also track their data over time through a mobile application or websites [5]. However, data collected by these systems should be relevant, understandable, and pervasive for the purpose of providing an accurate measurement and better health outcome.

### 1.1. Statistical Data about Obesity in the World

The World Health Organization (WHO) [6] has published that each year overweight or obesity is the cause of 2.8 million people's death and 35.8 million people (about 2.3%) of disability-adjusted life year (DALY).

**Figure 2:**
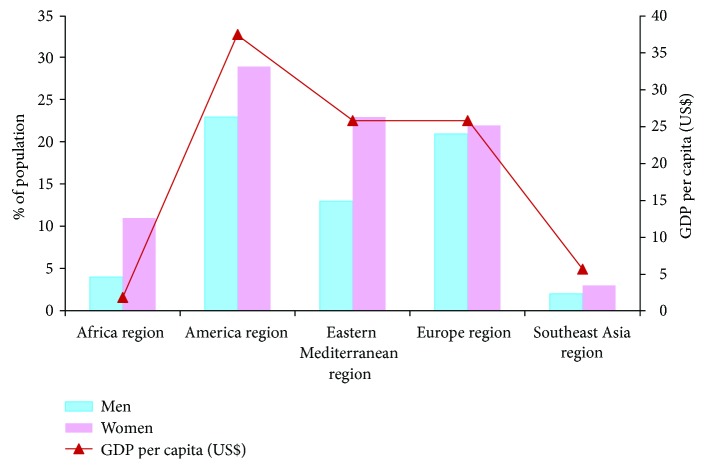
Percentage of obese population older than 20 years with a BMI higher than 30 kg/m^2^ and its relationship with the GDP per capita.

The presence of overweight and obesity implies adverse metabolic effects on blood pressure, cholesterol, triglycerides, and insulin resistance. The risk of several types of cancer such as breast, colon, prostate, endometrium, kidney, and gall bladder is also increased by the increased body mass index. In order to achieve an optimum health, the average BMI for an adult should be in the range of 21 to 23 kg/m^2^, although the goal for individuals should be to maintain the BMI in the range from 18.5 to 24.9 kg/m^2^.  Figure 2 shows the percentage of obese population older than 20 years old with a BMI higher than 30 kg/m^2^ [7].

Although it is not fully demonstrated, there are indications that relate the index of countries' globalization with the levels of obesity. Social globalization measures factors such as lifestyles that promote unhealthy food and sedentary lifestyle. Economic globalization also has an influence on obesity, although this aspect of globalization may seem more logical, since it is related to the income of each person or the price of food, among others [8].  Figure 2 also shows the relationship between obesity levels and the value of the gross domestic product (GDP) per capita [9]; it is easy to see the economic factors.

Analyzing data of overweight and obesity (see Figure 2), it is easy to observe that the population distribution with this problem is not homogeneous around the world. While the population of American region with a BMI higher than 30Kg/m^2^ is about 23% in women and 30% in men, areas like the region of Southeast Asia are the lowest ones for both sexes. European region and Eastern Mediterranean region present very similar values for women (around 22-23% of total population). However, the values for men are very different. While European region is about 21% of people older than 20 with a BMI higher than 30 kg/m^2^, this value is around 13% for Eastern Mediterranean region. Finally, it is important to highlight that in Africa, Eastern Mediterranean, and Southeast Asian regions, women present roughly double the obesity of men.

### 1.2. Obesity and Related Illnesses

Obesity is defined as an overabundance of body fat. The body fat content can be measured by the BMI which is calculated as the percentage of weight (in kilograms) to height squared (square meters). From a clinical perspective, a BMI of 25–29 kg/m^2^ is named overweight; higher BMI (30 kg/m^2^) is named obesity. In order to measure obesity, physicians usually use several methods based on the calculation of the percentage of total body fat which includes bioelectrical impedance, skinfold thickness, and underwater weighing [10]. Obesity and overweight are often a very important conditioning factor in chronic diseases such as asthma or heart disease. Obesity is also considered one of the triggers of other diseases such as hormonal problems, poor blood circulation, or respiratory insufficiencies, among many others. All this influences the individual's quality of life and longevity. Obesity also imposes a significant cost to healthcare systems. Some chronic diseases are directly associated with obesity like the sleep apnea, some type of cancer, atherosclerotic cardiovascular disease (ASCVD), and type 2 diabetes [10, 11], among others. For a healthy lifestyle, the Center for Disease Control and Prevention (CDCP) recommends the practicing of PA during 60 minutes per day for children and 150 minutes per week for adults. This routine should include bone-strengthening, muscle-strengthening activities 3 times a week. To achieve these goals, new wearable technology trackers have been developed. These gadgets can quantify the users' PA in an effort to raise the level of physical activity guidelines compliance [12]. PA is playing an intrinsic role in combating obesity and achieving a healthy lifestyle.

Diseases such as metabolic syndrome diabetes mellitus, cardiovascular disease, nonalcoholic steatohepatitis (fatty liver), gallbladder disease, gastroesophageal reflux, obstructive sleep apnea, reproductive system disorders, many cancers, and osteoarthritis as well as social and psychological problems [13] are associated with obesity. Many studies showed that high percentage of diabetes risk can be attributed to excess weight. This risk of disease increases 5-fold for people with a BMI of 25, 28-fold for a BMI of 30, and 93-fold for people with a BMI of 35 and greater. Furthermore, a waist circumference greater than 40 inches significantly increases the risk of diabetes, even after controlling for BMI [14].

Recently, the relationship between overweight and obesity to cancer has been studied by Calle and Thun [15]. The study showed that in obese individuals the relative risk of colorectal cancer is ranged from 1.5 to 2.0 in men and from 1.2 to 1.5 in women. Confirmed breast cancer studies indicate that the risk of developing the breast cancer in obese postmenopausal women increases by 30–50%. As a summary, the estimations showed that there are approximately 90,000 deaths per year due to cancer underlie obesity. This is an important indicator of how serious this problem is in terms of rising morbidity and mortality in the population. Prevention of obesity could thus notably minimize the incidents of cancer in both men and women as it is evaluated that this condition could account for 20% of cancer deaths among women and 14% among men [13].

Breathing difficulties during sleep are a common result of obesity. Some obese suffer from different changing during sleep such as having low oxygen saturation, snoring, and sleep apnea. An obesity study done in Sweden reported that over 50% of the men and 33% of the women with a BMI 35 suffer from snoring and sleep apnea. These sleep changes seem to increase the risk of myocardial infarction and stroke.

### 1.3. Summary of Contributions and Motivation

As far as we know, there is no work where the main parameters used to measure overweight and obesity have been analyzed. We have not found any study or survey where the most recent proposals and medical instrumentation to diagnose obesity are compared. We have seen some proposals with interesting ideas, but none of them are able to combine physiological and cognitive aspects to improve the results and evolution in the process to combat obesity and overweight. For this reason, this review presents a study of the main systems to diagnose the degree of overweight and obesity. It also explains the causes and consequences of suffering overweight and obesity and how the diseases related to obesity influence the individual's quality of life. Furthermore, the paper shows several of the most used mobile applications and wireless body area networks (WBANs) used to measure body parameters and to quantify the obesity degree. Finally, we draw an intelligent architecture that considers both, physiological and cognitive aspects to help reduce the degree of obesity and overweight.

The rest of this paper is structured as follows.  Section 2 analyzes the main parameters used to measure the degree of overweight and obesity and to define the body composition. The obesity and fitness monitoring systems to control obesity are shown in Section 3.  Section 4 shows some interesting proposals focused on WBAN systems for monitoring the physical activity and vital signs of people monitoring. As a summary of our study, in Section 5, we propose a new architecture that combines the physical aspects and the cognitive set point for controlling the body weight. Finally, Section 6 presents the conclusion and future works.

## 2. Causes of Obesity and How to Measure It

This section highlights the commonly applied obesity measurement parameters that are used for body composition assays to identify subjects before they have reached the extreme obesity range.

### 2.1. Causes of Obesity

There are several causes that cause overweight and obesity [16]. Experts agree that the main cause is the lack of energy balance. In order to have an energy balance, the energy consumed in food must be equal to the energy expended. The energy we eat is the amount of energy or calories we get from food and beverages. The energy expended is the amount of energy the body uses in functions such as breathing, digesting food, and staying active. There are other factors that also contribute negatively to obesity and overweight:

- An inactive lifestyle: many people do not stay physically active. Many people spend hours in front of the television and the computers at work, doing homework or a hobby. People use the car to go from one place to another instead of walking. Technology and facilities have reduced physical demands at work and at home; it has also been influenced by the lack of physical education lessons for children in schools. The less active the people are the more likely they are to gain weight because they do not burn the calories they consume in food and beverages. An inactive lifestyle also increases the risk of suffering coronary artery disease, high blood pressure, diabetes, colon cancer, and other health problems.
- Environment: our environment does not always contribute to having healthy habits. In fact, it stimulates obesity. This is due to reasons such as working days that do not allow leisure activities and free time for practicing exercise or lack of access to healthy food.
- Genes and family history: overweight and obesity tend to be hereditary. The chances of being overweight are greater if one or both parents are overweight or obese. Children adopt the habits of their parents. Thus, a child of overweight parents, who consume foods high in calories and are less active, will probably become overweight as well. On the other hand, if the family adopts healthy habits regarding food and exercise, the chance that the child will become overweight or obese decreases.
- Health problems: some hormonal problems can cause overweight and obesity, including hypothyroidism, Cushing's syndrome, and polycystic ovarian syndrome.
- Medicines: certain medicines can cause weight gain. These include some corticosteroids, antidepressants, and anticonvulsants.
- Emotional factors: some people eat more than usual when they are bored, angry, or stressed. Over time, overeating leads to weight gain and can cause overweight or obesity.
- Smoking habits: some people gain weight when they stop smoking. The main reason is that foods often taste and smell better. Another reason is that nicotine increases speed in the body that burns calories so the person burns fewer calories when she/he gives up smoking. However, smoking poses a serious health risk and quitting is more important than the possibility of gaining weight.
- Age: as we get older, we tend to lose muscle mass. Loss of muscle mass can slow down the rate at which the body burns calories. If people do not reduce their calorie intake as they age, they can gain weight.
- Pregnancy: during pregnancy, the woman gains weight to support the growth and development of the baby. After delivery, some women cannot lose weight. This can lead to being overweight or obesity, especially after several pregnancies.
- Lack of sleep: the modern lifestyle encourages evening activities. The electric light, the television, and the computer offer the opportunity of entertainment during the night. However, school and work schedules have not changed and require individuals to wake up early. The immediate consequence of this situation is the reduction in the sleep hours. This phenomenon mainly affects children and teenagers who remain awake for long hours at night turning the decrease in sleep duration into a characteristic of their lifestyle [17] (Burkhauser and Cawley). A fundamental system in body repair is the circadian system that regulates the metabolic functions in the day and at night. During wakefulness, our organs are prepared for energy consumption, digestion, and nutrient utilization [18]. During the day, physical activity, energy wastage and food and water consumption predominate. During sleep, the body carries out the energy saving and storage, the digestive processes are reduced, but the processes of cellular repair as well as the rest and memory organization increase. During the night, the secretion of some hormones as melatonin or growth hormone contributes to the synthesis of cell repair proteins [19] (Leproult and Van Cauter). Sleeping is also necessary to carry out functions of memory formation and mental organization, muscle relaxation, and energy saving. The possible relationship between poor sleep and obesity can be explained by several approaches. For example, night work also promotes a reduction in the quantity and quality of sleep along with increased activity at night. In night workers, there has been a higher prevalence of overweight and obesity than in the general population, with a high predisposition to metabolic diseases, among others. Being awake at night is a sufficient stimulus to motivate the nocturnal ingestion of food, that is, 75% of the food is consumed during the day [20] (Wu et al.). Research has shown that lack of sleep increases the risk of obesity. People who sleep fewer hours also seem to prefer foods that contain more calories and carbohydrates, so they can overeat, gain weight, and become obese. Sleep maintains a healthy balance of the hormone that does not make us hungry (ghrelin) and that does not make us feel full (leptin). When a person does not get enough sleep, the concentration of ghrelin increases and the leptin level is reduced. Then, we are hungrier than when we have rested well. Sleep also affects how the body reacts to insulin, the hormone that controls the concentration of glucose (sugar) in the blood. Lack of sleep causes higher blood sugar concentration than normal, which can increase the risk of suffering diabetes.

### 2.2. Anthropometric Measurements

Anthropometry refers to the measurement of the human individual. Anthropometric measurements are systematic measurements of the size, shape, and composition of the human body. There are several factors, but the most important ones are used to measure and determine the obesity and overweight level:

- Body mass index (BMI): anthropometry involves measurements of weight (*W*_t_), height (*H*_t_), circumferences and lengths at different body regions, and skinfold thickness (SF). It is inexpensive, easy, and fast and tends to have a small percentage of error in adults [21]. Commonly, total body fat is measured by body mass index due to its simplicity and inexpensive way to calculate and correlate with an adult's total body fat, and it is defined as (*W*_t_/*H*_t_^2^). Gender, age, race, and smoking status are all factors that influence the relationships between BMI and death or major comorbidities [22]. In general, these relationships will be low in an individual with BMI range from 18.5 kg/m^2^ to 24.9 kg/m^2^ and increase with the range from 25 kg/m^2^ up to 40 kg/m^2^.  Table 1 presents weight classifications by BMI to classify obesity. We should take into account that the BMI is based on height and weight for measuring overweight and obesity. For this reason, the results in body weight classification can be erroneous for people who have a greater proportion of bone mass and muscle tissue. Another BMI drawback is that it does not reflect body fat distribution (central trunk versus hips and thighs) which is associated with metabolic disturbances and cardiovascular risks [21, 22].
- Waist circumference, neck circumference, and intra-abdominal obesity measurements: the overall body fat is not the only concern; the distribution of fat matters too. Abdominal fat mass or also referred to as central or visceral obesity is a type of obesity that increases the risk for metabolic derangements coronary heart disease, type 2 diabetes, and cardiovascular disease [23]. Several measuring techniques for intra-abdominal fat content which can be accurately performed include computed tomography (CT), magnetic resonance imaging (MRI) which is also called imaging techniques [24], or waist circumference techniques. In contrast with BMI, the waist circumference and waist-to-hip ratio (WHR) are indirect methods that can be used to measure the fat distribution and abdominal obesity. It is one of the main diagnosis components of metabolic syndrome, and it is the most commonly used abdominal obesity indicator. Waist circumference is the cheapest method and can serve as an indicator for the relationship between waist circumference reduction and anticipated improvements in metabolic parameters. Another relatively new technique called neck circumference which is an economical and practical measure is known as the suitable marker for upper body obesity. Neck circumference correlates positively with cardiovascular risk, metabolic syndrome risk, pregnancy-induced hypertension, and high blood pressure in children. In comparing with BMI and waist circumference, neck circumference is considered as a stronger, valuable, and noninvasive diagnostic marker for indicating the decreased serum HDL cholesterol and elevated serum triglycerides in both sexes [25].
- Skinfold- (SF-) based technique: one of the most commonly used anthropometric methods for estimating fat percent is the skinfold thickness method. SF is based on the hypothesis that the thickness of the subcutaneous adipose tissue reflects a constant proportion of the total fat mass. It can be applied to various areas of the body such as the trunk, chest, triceps, abdominal, thigh, suprailiac, and subscapular using inexpensive mechanical calipers that provide significant and reliable information for estimating the subcutaneous fat layer [26]. SF method provides a noninvasive, quick, and cost-effective means of estimating body composition. However, the accuracy of skinfold technique has been questioned for years due to the high variation of skinfold data which depend on the operator. At best, this technique provides an acceptable measure of the subcutaneous layer covering the body. Currently, over 100 prediction equations of SF have been developed that are different in terms of gender, ethnicity, and age of the group been checked. An accurate SF assessment can be done in just a matter of minutes, any place or time. Alternatively, the use of mechanical calipers has been replaced with high-technology techniques for automatic and operator-dependent errors measurement of the subcutaneous fat layer. However, these technologies still have limitations linked with extrapolation to the body's total fat mass.

Table 2 summarizes the main anthropometric measurement methods, the technology used, and their advantages and disadvantages.

The errors associated with anthropometric methods typically range from 3 to 4% and can be larger if nonadequate equation or measurement technique is applied. There are other more accurate techniques for estimating the percentage of body fat, but they must be applied in a laboratory and they are commonly used for research or validation of newer techniques [27].

### 2.3. Machine-Based Measurements

Densitometry techniques are used to measure body volume from density which is the mass divided by the volume. Underwater weighting (UWW) and plethysmography are typically the two main densitometry methods available at laboratories and clinics throughout the country.

**Figure 3:**
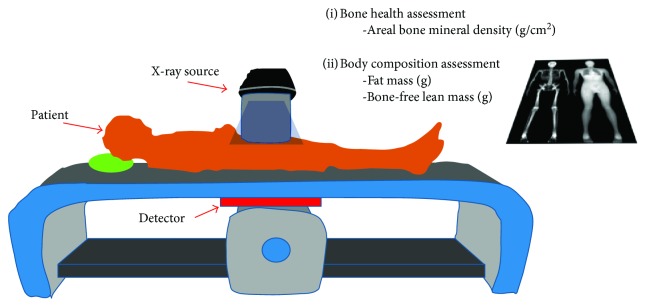
DEXA scanner.

**Figure 4:**
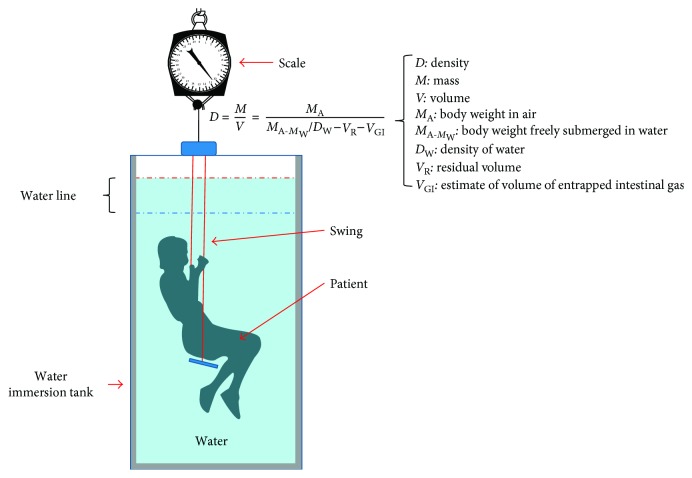
The underwater weighing technique.

**Figure 5:**
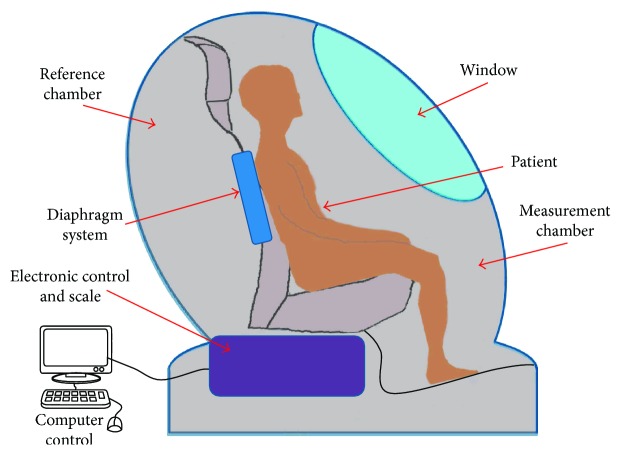
ADP camera.

**Figure 6:**
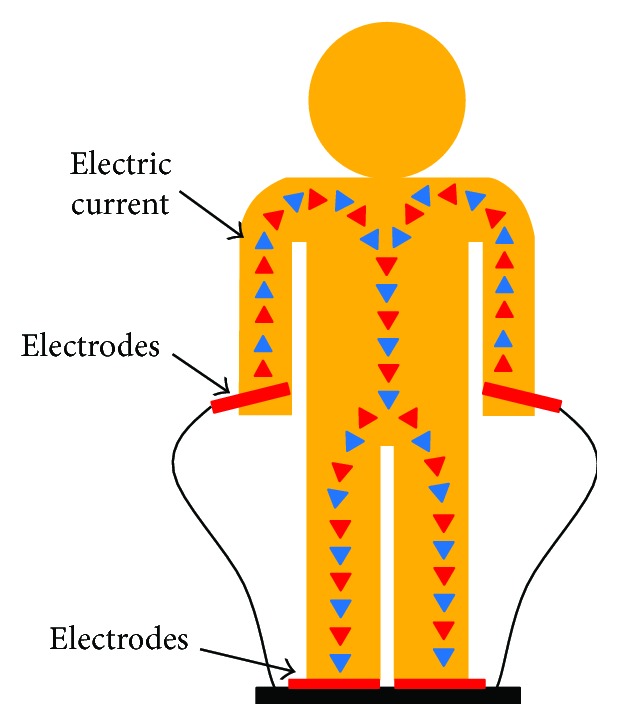
Schema of BIA method through a person.

**Figure 7:**
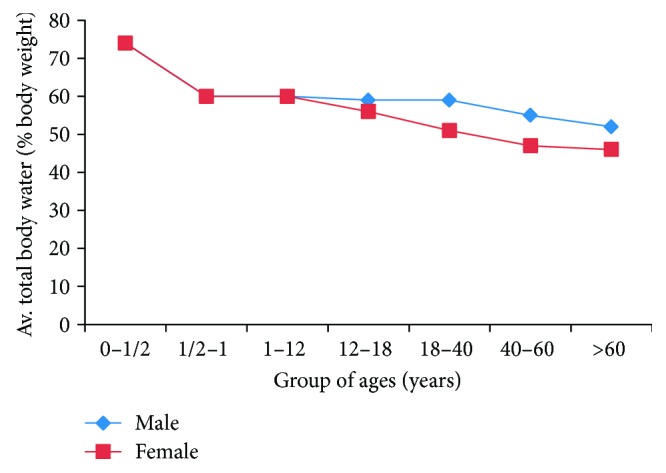
Average TBW as a function of gender and ages.

**Figure 8:**
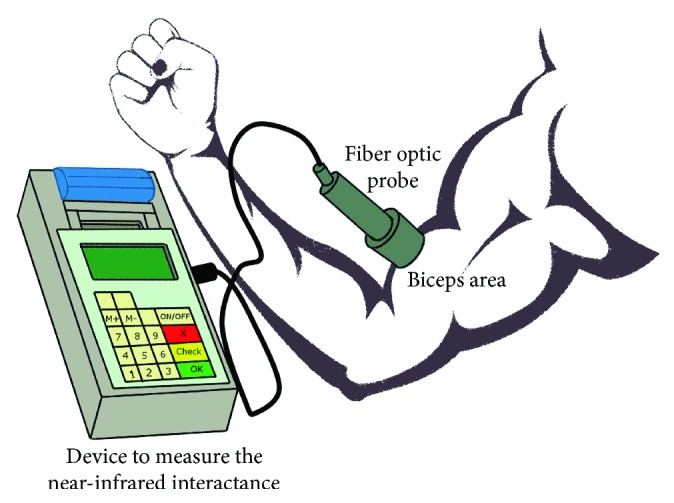
Process to measure the fat density with the near-infrared interactance method.

- DEXA (dual-energy X-ray absorptiometry): a DEXA scan [28] is used for measuring bone mineral density inside body composition by exposing the body to different intensities of X-ray beams. The machine arm passes through the patient's body parts individually and emits a low- and high-energy X-ray beam. The absorbed beam will be measured, and the technician can obtain readings for bone mineral density, fat mass, and lean body mass. The advantage of DEXA scans is incredible accuracy at evaluating body composition. A DEXA scan simply requires lying on a table for a few quick, dry, and painless minutes whereas the hydrostatic weighing method requires being dunked under the water. Acquiring a DEXA scan usually requires taking an appointment with a medical professional in a clinic.  Figure 3 shows a DEXA scanner and its main functions.
- Underwater weighing and air displacement plethysmography (ADP) methods: underwater weighing and air displacement plethysmography (ADP) are considered undisputed reference methods for measuring fat and fat-free mass, and they are based on body density. The underwater weighing method (see Figure 4) is underlying on the fact that fat floats on water and generally in less-dense liquids. So estimating the volume of displaced water by the body and the variance in body weight under normal condition and in water permits an evaluation of total body density. This method is highly accurate and precise and only used as a gold standard. However, it is costly and difficult to implement in clinical practice. ADP (see Figure 5) is an accurate and gold standard method that also utilizes the same underwater weighing method principle, except that the criterion underlying this technique is the air displacement, rather than water displacement. The ADP method is highly expensive and demands special apparatus and the use of compression underwear. There are several alternative reliable measurement methods to estimate the body fat mass which include dual energy X-ray absorptiometry (DEXA) and computed tomography (CT). These techniques are applied to validate newer and too costly methods to be used routinely in clinical practice [26].
- Bioelectrical impedance analysis (BIA) and total body water (TBW) estimation: bioelectrical impedance analysis (BIA) is a fast, noninvasive, and nonintrusive fat-free body mass (FFM/FFBM) and TBW estimating technique. A common technique in this field is BIA which grounded on the premise that when an electrical current is passed through a certain region of the body, the voltage drop between two electrodes is proportional to the fluid volume of that body region. Bioelectrical impedance scales range from the simple (a normal scale with electrodes under each foot) to the complex (a scale that has handholds with additional electrodes). These scales work by sending tiny electrical impulses through the body and measuring how quickly those impulses return. Since lean tissue conducts electrical impulses quicker than fatty tissue, a faster response time is correlated with a leaner physique. Most of these scales combined with built-in body composition features can quickly generate and track fat percentage alongside bodyweight. The advantages of bioelectrical impedance instruments are relatively inexpensive (compared with a mass spectroscopy instrument). Their operations do not need highly trained personnel, and they have good reproducibility results in a matter of seconds [29, 30]. The BIA measurement (see Figure 6) is applied by appending a pair of electrodes at the wrist and at the ankle for the purpose of allowing a weak alternating current (800 milliamps) to be passed through the body. During the stability of the current, the voltage drop and resistance (*R*) will be calculated. To assess the volume of TBW, the following assumptions are used: (1) the whole body performs like a cylindrical conductor, (2) the subject's height is proportional to the conductor's length, and (3) the reactance component of the voltage signal can be ignored. Under these circumstances, the impedance index calculated as *H*_t_^2^/*R* is supposed to be proportional to the TBW volume. To obtain an accurate, easy, and fast TBW measurement, activities such as moderate to vigorous exercise, excessive sweating, or consumption of excessive alcohol should be performed within 4 h before the assessment. As Figure 7 shows, the TBW is affected by the gender and ages. Comparing this method with the skinfold method, the predictive accuracy of BIA is relatively similar to that of the skinfold method but BIA is also used to measure body water's resistance and it can estimate obese body composition. One advantage of BIA is that it does not need high technical skills to perform the impedance measurement. However, it is influenced by food intake, hydration level, exercises, menstrual cycle stage, and skin temperature. The actual measurement procedure of BIA for the subject is relatively easy and can only take a few minutes.
- Near-infrared interactance (NIR) method: several research studies have shown that the NIR method is a useful method for body composition assessment and many commercial instruments have been designed to evaluate predictively the body composition in children and adults. The term NIR refers to the response that the superficial layers of the skin have when an infrared light is emitted on it, such as changes in pigmentation. This method allows measuring parameters such as blood sugar or pulse oximetry, among others. The NIR method is based on the principles of light absorption, reflectance, and near-infrared spectroscopy. In order to determine the body fat composition, the physician uses a spectrophotometer with a hand-held fiber optic probe (see Figure 8). The probe is pushed against the biceps muscle and emits an infrared light. This light passes through the fat and muscle tissue to reach a bone and reflects it back to the probe. To calculate the fat density, the results are introduced into predictive equations that consider parameters such as height, weight, and body type. The final results give an estimation of the overall body fat percentage. This method is not as accurate as DEXA scanning or hydrostatic weighing, but it is slightly better than the results offered by BMI [26].

Table 3 shows the availability, cost, and reliability of machine-based and anthropometric methods.

## 3. Obesity Control: Obesity and Fitness Monitoring Systems

Toward a good lifestyle and free obesity risks, researchers examining low physical activity and sedentary and food intake behavior as potentially independent risk factors for chronic disease morbidity and mortality have expanded rapidly in recent years. Several subjective and objective methods that are suitable for use in population-based research have been developed, while the new generation of smart sensors and ubiquitous monitoring systems offers unique opportunities to measure real-life environments, mobility, physiological responses, and physical activity. Objective techniques use current wearable or body-fixed motion sensors, which include switches, pedometers, actometers, goniometers, accelerometers, and gyroscopes, video recording system, optical systems, and mechanical systems for assessing PA, food intake, and sedentary time.

PA is a multidimensional behavior categorized by various aspects, such as intensity, frequency, duration, and type. It is the key solution for obesity and other health complications such as glucose tolerance and hypertension [31], while sedentary behavior is typically described as activity demanding low EE levels that arise while sitting or lying down. Sedentary behavior has been the subject of growing epidemiological research in recent years [32, 33].

This section presents a narrative overview of the PA, food intake, and sedentary behavior measurement literature.

### 3.1. Sensor-Based Monitoring Devices

Several subjective and objective methods have been utilized in to report a user's PA. Subjective methods are considered very popular in large-scale studies due to their relatively low cost and include the direct observations, activity logs, diaries, questionnaires, and recall. However, the accuracy of subjective methods is based on a participant's ability to recall the frequency of purposeful activity period and they cannot explore the diverse quantitative aspects of PA [34]. Objective methods such as pedometers and accelerometers provide a reliable PA and sedentary time estimations because they rely on measuring physiological outcomes such as metabolic cost, body temperature, and heart rate or biomechanical effects, like displacement and acceleration related to PA and sedentary time in free-living conditions. However, these methods are relatively applicable in large studies due to the need of expensive tools. The gold standard methods that are used to assess the PA are direct/indirect calorimetric and doubly labeled water methods. They are often used as tools to validate newer PA assessment tools [35]. Calorimetric methods are also limited to clinical and laboratory use with an expensive cost [36]. This subsection discusses the main methods used to monitor the PA and sedentary behavior.

- Pedometers: mechanical pedometers also referred to as “step counter” are becoming more popular and commonly used for PA monitoring in the laboratory and daily life. Pedometers are based on switching mechanisms such as a spring-loaded mass mechanism for detecting the obvious impacts generated by steps during locomotion [37]. Pedometer estimates the energy expenditure and the distance walked by registering the number of steps during motion. These devices are widespread, and it is important that they be accurate since this can have a significant impact in determining the number of steps per day that an individual takes. Several studies have tested the reliability and accuracy of pedometer devices for estimating PA and predicting activity energy expenditure under both free and controlled living conditions for greater BMI and waist circumference. These studies showed that spring-levered pedometers reduce the step count accuracy. Another type of pedometers called inertial pedometer which is based on a piezoelectric sensor is capable of measuring the variation of the applied acceleration forces during movement, and it is more accurate in PA measurements for obese patients. However, inertial pedometers are 10 times more expensive than spring-levered pedometers [34, 38]. The main drawbacks in pedometers are the insensitivity to nonambulatory activities and the inability to reflect movement intensity. These facts give as a result inaccurate estimations of energy expenditure. Alternative solutions for accurately measuring physical activity are the use of optical systems, magnetic systems, and video-based recording systems. However, optical and magnetic systems are costly and need environment setting and sophisticated instrument. Video-based monitoring systems collide with privacy concern making them impractical routines for the free-living environment.
- Accelerometers: accelerometers have become the alternative noninvasive and easy to use solutions for measuring PA, sleep, sedentary behavior, and total daily energy expenditure (TDEE) [5, 39]. They are also low-cost and small-sized devices with a robust design able to measure the body movement in terms of acceleration. Accelerometers can help in monitoring and estimate the intensity and the duration of the user's PA over time. An accelerometer device consists of piezoelectric sensors that can detect acceleration in all dimensions. Accelerometers can also contain an internal memory to store processed data before it can be transferred to the user's cell phone or web application for further analysis. The accelerometer can measure the user's PA in many ways through either a digital counter or an algorithm, among others. Accelerometers have several recognized drawbacks among which included their inability to failure to accurately measure activities such as carrying objects, cycling, or lifting [39]. They are also unable to correctly differentiate between standing and sitting situations. Furthermore, accelerometer devices are suffering from lack of standardization because the outputs are based on the algorithm that is used for interpreting collected data and the subsequent cut-point values.

### 3.2. Commercial Sensor-Based Monitoring Devices

There exists a wide spectrum of sensor-based monitoring devices that can help a user in monitoring PA, controlling weight, and tracking energy expenditure (calorie burn). These devices are varying in terms of features and functions, but they all attempt to provide some degree of accuracy, lower cost, mobility, and easy usage. Some examples of these devices are the Fitbit [40], the DirectLife (DL), the BodyMedia FIT (BMF), the NikeFuel Band (NFB), the Basis B1 Band (BB), and the Jawbone Up (JU) Band. Several studies have been performed to examine the accuracy and reliability of these accelerometers.

- BodyMedia armbands: they are also known as the SenseWear Armband device. The BodyMedia armband was originally developed by BodyMedia Inc. (Pittsburgh, PA), and it was available on the market since 2001. The current generation of this device is highly attracting the consumer sector because it can facilitate consumer self-monitoring and weight management. BodyMedia armband is based on multiple sensors that work concurrently to obtain a real-time activity context of the user. In contrast, these sensors provide sensitive estimations for the user's physiological parameters. The current consumer version utilizes four types of sensors: a three-dimensional accelerometer to track the user's upper arm movement and to offer body position information; a synthetic heat flux sensor which is utilized to measure the amount of dissipated heat by the body; a sensitive thermistor to measure skin and armband-cover temperature; and finally, a sensor to measure the galvanic skin response (GSR) [36, 41]. The device offers a watch interface, and it is able to wirelessly connect with mobile apps for a monitoring purpose. The BodyMedia armband has rechargeable batteries that can remain for 2 weeks [5]. Through the user's armband, the BodyMedia armband system can obtain physiological data which can be analyzed, interpreted, and stored within the device. With the aid of a built-in onboard algorithm, the device can provide a real-time estimation of the parameter of interest such as the number of steps and calories burned. Obtained data can be viewed on the user's cell phone, on a BodyMedia itself, or on the user computer when these data have been transferred to the computer through a USB or wireless technology. The company released other versions of BodyMedia armband devices that perform the same tasks but differ in some features such as increased memory capacity or Bluetooth wireless interface. Available versions include the BodyMedia FIT and the Bodybugg from 24 Hour Fitness. The BodyMedia FIT program is developed to assist people to lose weight. However, to correctly work and interpret the gathered data, users need to enter their profile and daily meals which can be transformed by Withings SA in June 2013 [42].
- ActiGraph GT3X: in mid-2009, ActiGraph (Pensacola, FL) released the GT3X that contains the ADXL335 accelerometer manufactured by Analog Devices. The ADXL335 is a 4 × 4 × 1.45 mm triaxial capacitive microelectromechanical system (MEMS), and it also contains a filter and the analog to digital converter (ADC). The GT3X is an exclusive research device used for testing the validity of most new PA trackers. It is capable of measuring static accelerations and provides inclinometer outputs. Users can wear this accelerometer on their hopes for measuring PA and active time under free-living conditions [39]. The device is able to distinguish whether a user is not wearing it because it uses vector magnitude data from the three axes and assigns a number for different situations; that is, it is not worn (number 0), the user is standing (number 1), the user is lying (number 2), and the user is sitting (number 3).
- Withings Pulse was developed by Withings SA in June 2013 [39] as a health tracker that supports Wi-Fi technology. It is capable of measuring pulse and heart rates as well as record sleep time in terms of optimal sleep hour's percentage. The Withings Pulse can act as a tracker for daily life activity or fitness. The device provides health statistics that include active calories, step counting, and distance traveled. Several versions are Pulse Ox, Activité Pop, Activité Steel, and Activité [43, 44].
- Fitbit was released by Fitbit Inc. It is a thumb-size activity tracker designed to be worn 24 hours a day. Fitbit can be used as a multitasking device. Users can use it during the day to record their steps and for tracking their sleep process at night. Due to the Fitbit wireless connectivity, users can access their recorded data through three ways, that is, accessing data from an iPhone application, seeing data in the Fitbit itself, or visualizing data via web application [41]. However, users require inputting the food information via an online questionnaire. Some versions currently available are Fitbit Ultra (FU), Fitbit One (FO), Fitbit Zip (FZ), Fitbit Charge, and Fitbit Flex, among others [43].
- Fitbit One is a clip-based device used to track steps, calories burned, distance, sleep, and floors climbed. The device can be used to track multiple measures of PA such as the number of steps and distance traveled. It is also able to wirelessly upload activity data to a website for the purpose of tracking activity levels over time. This real-time data connectivity feature attracts researchers and patients alike for tracking PA such as walking. Studies have been made to evaluate the validity or reliability of the Fitbit One device.
- Fitbit Zip consists of a triaxial accelerometer that performs the same Fitbit One tasks (measuring the number of steps, distance traveled, and calories burned) with some new features. The Fitbit Zip is smaller than previous versions (35.6 × 28.9 × 9.6 mm). It is slightly less expensive (around $60), and finally, it presents an expanded battery life of approximately 4–6 months. Beside basic tasks (step count, distance traveled, and calories burned), the Fitbit Zip is also capable of measuring multiple variables such as minutes spent in light and vigorous and moderate physical activity levels, among others [35]. The Zip screen has an indicator that displays basic data such as steps taken, calories burned, and distance traveled. The Zip is considered a hassle-free device, but it requires minimal technical knowledge for setting up and operating. For the purpose of data analysis and display, Zip device does not need any specialized computer hardware or software. It is based on web-based software wherever an internet connection is available. If no internet connection is available, the Zip can sync to an Android smartphone or iOS and show the data via the free Fitbit app [35, 44].
- DirectLife is a small-size and lightweight monitoring device developed by Philips Lifestyle Incubator for enhancing the possibility of wearing smart devices and minimizing the monitoring interference with spontaneous activity conduct [45]. It is composed of a triaxial accelerometer based on the Tracmor [46], and it is designed with an internal memory that allows the user to store data for a long period (approximately 22 weeks). It also has a battery with autonomy of 3 weeks and is waterproof up to 30 m depth. Participants can track their EE estimation data through a personal website which provides them with tips, activity ideas, and statics [5]. Users of the DirectLife can recognize the amount of exerted PA per day as specified by preset goals through the device monitor which contains an indicator bar of light-emitting diodes [45].
- A Jawbone UP Band is a device manufactured by Jawbone in November 2012 [46] for a user's sleep and physical activity pattern assessments. It is a wristband device based on a three-dimensional accelerometer. Due to its accuracy, it is considered one of the top fitness trackers that can accurately track the steps taken, calories burned, distance traveled, active minutes, and sleep disorders. The Jawbone UP4 is the new commercial version that is able to continuously perform the heart rate tracking at any time during the day. The device is also capable of synchronizing data with social media and mobile applications, and it can send idle alerts in the form of vibrations when the user is sitting for a long time. This feature is a great way to keep users stay active throughout the day. Another handy feature that Jawbone UP offers is the respiration and sweat tracking which gives users insight into how they are active on a daily basis [44, 47].
- The Misfit Shine was released in August 2013 by Misfit Inc. It is promoted as a companion to Android and iPhone App for fitness tracking purpose. It is an activity- and sleep-tracking device that allows users to track their movements and activity levels. The Misfit Shine also tracks hours of light and sleep quality as well as daily activities such as step counting, distance traveled, and calories burned [39, 44].

### 3.3. Food Intake (FI)

The primary key to maintain a healthy lifestyle is keeping a balance between energy gained and energy expended. Objective methods such as a questionnaire and recall methods have failed in providing a good estimation for intake history. Miniature sensor devices can easily help in detecting ingestion events such as capture timing, a microstructure of food intake episodes, energy contents of food, or characterization of the rate of ingestion, duration, ingested mass, and nutrition, for further characterization of ingested foods [2, 11]. Most of these systems combine multiple sensors for monitoring physiological changes and organ movements linked with food intake and signal processing and/or pattern recognition algorithms for determining how and when food is consumed. In this regard, several methods have been proposed for assessing free-living food intake including observation, estimated records, weighed food records, food-frequency questionnaires, multimedia diaries [48], and food recall methods [49].  Table 4 shows a summary of the main methods to measure the food intake [11].

However, the aforementioned methods have some limitations. On the one hand, these methods are not capable of specifying individual eating episodes. On the other hand, they are considered inaccurate methods due to subjects tending to misestimate food consumption [50].

Recently, researchers have proposed the use of chews and swallows as indicators of FI for objective monitoring of ingestive behavior (MIB) [50–52]. These proposals monitored the chews and swallows during the period of talking, quiet resting, and meals. The outputs of this monitoring along with other derived metrics were calculated to build prediction models for food intake detection, food mass estimation, and differentiation between solids and liquids. On the one hand, swallowing-based food intake monitoring systems are relying on the monitoring of electrical muscle activity, sounds, larynx motion, and variations in electric impedance across the neck at larynx level. On the other hand, chewing food monitoring is relying on monitoring the electrical activity of jaw muscles, sounds, and variations of jaw shape [11, 12, 52, 53].

Hand-to-mouth gesture or “bite” [53] monitoring is also involved in the development of food intake systems which give a motion indicator for food intake and ingestive behavior. To achieve this type of monitoring, standard inertial sensors (e.g., a gyroscope) can be utilized to detect wrist motion [54]. This section will discuss some proposals of FI and MIB systems.

Acoustic-based food intake detection was proposed by Päßler et al. [55]. It is composed of a microphone and ear-pad able to detect FI activity. The system can detect generated characteristic sounds during chewing and swallowing of food through the use of an in-ear microphone. To detect FI activity from the acoustic signal, two signal processing algorithms were built. The results show a high detection accuracy of approximately 83.3%. Another acoustic-based system that uses a wearable ear-pad sensor was proposed by Amft [56]. It is based on acoustic properties extracted from the microphone (air-conducted vibrations) to classify the sound of chewing during the food intake. The system proofed 86.6% accuracy for detecting four types of food using a pattern recognition classification algorithm. However, the experiments showed that it is very hard to use acoustic properties in detail food classification due to environmental noise. Also, these sensors can only work with restricted types of food.

Other groups and individual models have been created by Lopez-Meyer et al. [57] and Sazonov et al. [58] with the aid of swallowing information. These models can detect FI periods; however, the need for individual calibration remains a challenge. A solution for the acoustic problem is proposed by Amft and Tröster [59] which use electromyography sensors to detect swallowing action. However, this method is not convenient for long-term monitoring because it requires the user to wear a sensor collar around the neck.

Liu et al. [12] also proposed an intelligent food intake system for automatic eat activity detection. It is made up of wireless and a lightweight acoustic sensor that is combined with a miniature camera and in-ear microphone. The sound from the microphone is used in the process of sound feature extraction for the purpose of detecting chewing activity. When chewing activity is detected, the camera will be triggered to capture realistic snapshots of the food. The captured images are consequently added to a file which includes a series of food images and time stamps to outline consumption history.

A food intake detection scheme that is based on pattern recognition of jaw motion classification was developed by Fontana and Sazonov [60]. The system collects chewing signals during several daily living activities to add more variability, and it implements linear and radial basis function (RBF) and support vector machine (SVM) models by using time and frequency domain features. Experimental results showed that this classification model can discriminate FI periods with an accuracy of 90.52%.

## 4. Existing Systems

This section shows some important and interesting systems focused on people monitoring. Concretely, some of these systems are designed to monitor vital signs and are based on a WBAN composed of sensors for taking data of triaxial acceleration (ACC), electrocardiogram (ECG), blood oxygen saturation (OXI), and geographic position, among others. We can also find some mobile applications focused on measuring the physical activity.

### 4.1. Health-Oriented WBANs

A WBAN is defined as a set of low power devices such as microphones, headphones, or sensors used in the body that wirelessly communicates whit a central unit [61]. This network usually includes a smart and compact device, such as a smartphone or node [62]. The network may also consist of devices implanted in the body to control vital body parameters and movements. Data from these devices are usually transmitted to a base station, from which data can be sent, in real time, to a hospital, clinic, or caretakers. This type of data storage allows the development of intelligent databases and systems capable of making decisions efficiently. Because we are developing wireless communication networks, aspects such as the energy consumption [63] or the appropriateness of the communication protocols enhance the network performance [64].

These systems are able to collect patient data and improve the effectiveness of therapies. They can also support clinical decision-making and provide a comfortable and cheap communication channel between patients and healthcare staff. At present, there are many sensor networks whose data can be consulted through the Internet. The characteristics that pursue this type of networks are the following:

- Easy installation
- Self-identification
- Self-diagnosis
- Reliability
- Coordination with other nodes
- Software functions and digital signal processing
- Usage of standard network interface and control protocols

Recently, the popularity of WBANs for monitoring health status has been growing especially for obesity and weight control. Lee et al. [62] proposed a mobile phone centric WBAN called KNOWME which is currently deployed for pediatric obesity prevention and treatment. KNOWME consists of three Bluetooth enabled off-the-shelf sensors (ACC, ECG, and OX) and utilizes Nokia N95 mobile phone as a node. The features of this system are reducing sedentary behavior (lying down, sitting, and standing) and promoting PA in overweight Hispanic adolescents. KNOWME showed valid quantitative results from different mobile phones, and it is helpful for another kind of application that uses the sense-compute-communicate cycle.

Alrajeh et al. [63] proposed a WBAN framework for controlling obesity that consists of hardware and software architectures. The hardware architecture is responsible for the personal server and sensor nodes while the software architecture involves many modules such as a BMI calculator, calories calculator, and a calories consumption module, among others. Sensors are attached to the hands and feet, and a smartphone or personal computer can act as a server linked using a star network topology. The system used iMote2 sensors which have TinyOS and utilize IEEE 802.15.4 standard for the purpose of communicating with other sensor nodes. The system also utilized CSMA/CA with RTC/CTS mechanism for reducing the interference among nodes. However, the system results showed a significant difference in data delivery between when a user is in motion and when it is not.

Jovanov et al. [64] presented a WBAN based on a wireless sensor platform with a ZigBee-compliant radio interface and an ultra-low power microcontroller for monitoring PA and health status. This platform is equipped with accelerometers for motion monitoring and a bioamplifier for electrocardiogram or electromyogram monitoring. Software modules for onboard processing, communication, and network synchronization have been developed using the TinyOS operating system.

Otto et al. [65] designed a home-oriented WBAN system that continuously monitors user activity and heart rate. The WBAN sensors monitor the user's heart rate and locomotive activity and periodically upload time-stamped information to the home server. The home server integrates this information into a local database for the user's inspection, and it also forwards the information further to a medical server. The prototype can be used for ambulatory monitoring of patients undergoing cardiac rehabilitation or for monitoring elderly people at home by informal caregivers. There have been proposed five intervention strategies that may provide support at different disease stages (diagnosis, prevention, control, and treatment) and applicable to mobile health-oriented applications. These strategies include the health information tracking that involves the healthcare providers and take the advantages of entertainment for changing the user's habit, leverage social influence, and increased health information accessibility.

An effective epidemic control and source tracking through mobile social sensing over WBANs have been suggested by Zhang et al. [66]. The framework aimed to detect people's face-to-face social interactions. It employs Bluetooth as a first tier and acoustic meter detection technologies of mobile phones to catch the people's social interaction pattern.

Based on mobile technology, Pellegrini et al. [67] developed the E-Networks Guiding Adherence to Goals in Exercise and Diet (ENGAGED) program. ENGAGED is a randomized controlled trial (RCT) which uses a theory-guided technology that supported weight loss program. The ENGAGED program is designed to run on a smartphone (Motorola Droid™) and uses an accelerometer. The ENGAGED application contains the CalorieKing, a food database and a comprehensive nutritional source containing over 50,000 food entries, for self-monitoring daily dietary intake. It also uses goal thermometers to display the participant's goal and actually consumed the amount of calories and fat grams. The application also involves a Team tab that permits participants to view team members' adherence to self-monitoring and accelerometer usage. In addition, the Team tab contains a message board to facilitate peer-to-peer messaging communication among teammates.

An architecture of a WBAN for ubiquitous health monitoring has been proposed by Otto et al. [65]. This system can be applied for a quick response and high communications between patients and caregivers. The system comprises multiple sensor nodes for heart activity and body motion monitoring, a network coordinator, and a personal server which should run on a PDA or a personal computer. The sensor nodes can detect and transfer vital signs and relevant data to a personal server (PDA) through wireless personal area network (WPAN) implemented using ZigBee (IEEE 802.15.4) or Bluetooth (IEEE 802.15.1). PDA transfers this information to the medical server through the Internet or mobile telephone networks such as GPRS or 3G [68–70].

Metola et al. [71] present a scalable multidevice framework to increase self-awareness for health and disease prevention (FLES). It is a tool to simplify the deployment of medical check-ups and certain risk assessment components for avoiding various diseases including obesity. FLES enables integration of wireless sensors, health parameter control components, and disease risk assessment components to be deployed in Android-equipped instruments such as smartphones and tablets.

Table 5 compares the health-oriented WBAN solutions for obesity control presented in this subsection.

### 4.2. Health-Oriented Mobile Applications

Mobile sensing platform (MSP) has been used by different systems to transmit a list of activities such as walking, running, and cycling. Most of these mobile applications base their operation on the embedded sensors we can find in smartphones [72]. This subsection shows some of the most interesting health-oriented mobile applications we can find in the market.

UbiFit and Houston systems have been discussed by Klasnja et al. [73]. They present several systems that utilize MSP for encouraging regular physical activity (walking, running, cycling, using an elliptical trainer, and using a stair machine). The progress and performed activities can be monitored through a screen and a user-friendly interface. UbiFit application is connected to an MSP and back MSP transmits a list of activities with predicted likelihoods to the mobile device during the time of use.

Bond et al. [74] present the B-MOBILE application. It is specially designed to intervene on sedentary behavior in real time. The application is able to gather real-time accelerometer data from the smartphone and to calculate sedentary time by using validated algorithms. B-MOBILE permits monitoring the sedentary behavior goal setting, prompting, and feedback using an automobile dashboard that was visible when the smartphone display was active. The number of sedentary minutes is shown through the mobile dashboard which included a fuel gauge remaining until the next activity break. B-MOBILE uses two odometers that track the sedentary total number of minutes and accumulated active minutes during that day. If a user reaches the predefined limit of minutes spent in sedentary mode, the B-MOBILE app beeped and a reminder message appears to interrupt the activity.

Tsai et al. [75] present another mobile application called PmEB (Patient-Centered Assessment and Counseling Mobile Energy Balance). It consists of a client app running on the mobile phone, a server application, and a web interface that permits users for registering and personalizing the mobile client. The server application was built using Tomcat, and the client application using Java 2 Mobile Edition (J2ME). The main purpose of this app is to monitor real-time caloric balance.

SapoFitness is another mobile health (m-health) system designed for obesity prevention [76]. It is an intuitive and friendly application that utilizes mobile sensors such as accelerometers, camera, and GPS. SapoFitness aims to motivate users for losing weight, increasing their physical activity, and improving the balanced nutritional state. The application is able to access the personal health records (PHRs) through web services to the user food intake and physical activities data to determine his/her nutritional state. This application permits a continuous user monitoring irrespective of time and space.

Walkabout [77] is a walk-motivating application that proposes the user a set of motivating walking alternatives to ordinary routes by adding a social component. The application consists of a body sensor network (ECG and two accelerometers attached to the user's legs) and a mobile phone GPS sensor for enabling footpaths.

Another application focus on step counting called the StepUp which was present by Khalil and Glal [78]. This application automatically counts the number of steps walked by the user using sensor-enabled mobile phones. StepUp application aims to return the measured quantity of the user's daily activities and establish a healthy competition which works as a source of positive feedback. It also aims to surge the user's awareness and understanding of the significance of physical activities and facilitate the embedding of regular exercise into their daily life.

Some applications are focused on allowing users to retrieve their food intake history. In this regard, Zhu et al. [79] propose a prototype system that takes the advantages of the mobile built-in camera network connectivity to provide an accurate amount of daily food and nutrient intake by using an integrated image analysis. The system aims to utilize visualization tools with a nutrient database, permitting users to record their eaten food. Obtained images are used for estimating the consumed amount of food and nutrients.

Entertaining systems have been introduced encouraging people to live a healthy lifestyle. The mobile application/game called “Time to eat” is an example of this trend. It is an iPhone application presented by Pollak et al. in [80]. This application has been designed to promote and persuade children in practicing healthy eating habits. “Time to eat” game gives children the control of a pet that responds to photos showing the food it consumes. In addition, the pet sends an e-mail message as healthy eating reminders. The message is changed according to the day of the week. The game players must take and submit photos of their meal. Conversely, the users will then receive scores given by the pet, depending on the amount and healthiness of food eaten.  Table 6 shows a summary of health-oriented mobile apps that use MSP.

## 5. Architecture of a Smart System for Obesity Control

After analyzing the different parameters needed to quantify the degree of obesity and the existing applications and solutions, in this section, we describe how an intelligent system for controlling obesity should be implemented and which factors should be considered to achieve the best results.

**Figure 9:**
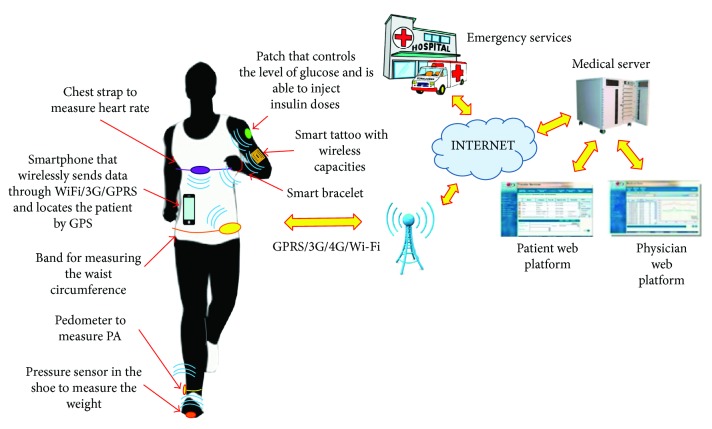
Architecture of a Smart System for obesity control.

An appropriate Smart System able to control and measure obesity should be composed by several sensors and small devices integrated as the elements of a WBAN (see Figure 9). Nowadays, we almost measure everything. On the one hand, it would be interesting to measure vital signs such as heart rate and breathing. Physiological parameters are also important, for instance, the waist circumference and the evolution of weight because they are associated with obesity. There could be other associated important diseases such as diabetes; this WBAN can integrate smart patch able to measure the level of glucose and inject insulin doses. Finally, this WBAN should be able to take measures about the PA through a pedometer and GPS location.

**Figure 10:**
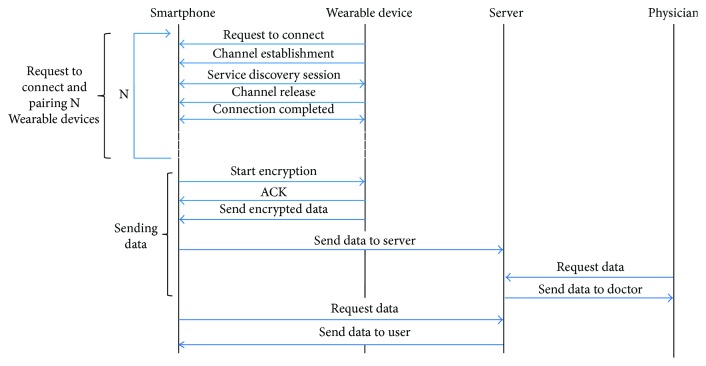
Message exchange for our architecture.

The data from all these sensors can be processed by a small microcontroller with wireless capabilities or a smartphone. Currently, we can provide a wireless connection to any corporal device thanks to the smart tattoos. These are small metallic circuits glued onto the skin with a microprocessor able to control, for example, some controls of a smartphone. The second important part of this architecture is the integration of this WBAN placed on the person's body in a more complex network that will store and process the data of the person, which could become a patient. The WBAN wirelessly transmits all captured data to a medical data server which would enable doctors and patients access to the medical health record generated from the data provided by this WBAN and information provided by physicians (see Figure 10). Finally, considering that these applications could be easily used for control and monitoring elderly and disabled people, it should integrate the ability to generate emergency calls to medical services when dangerous situations are detected.

**Figure 11:**
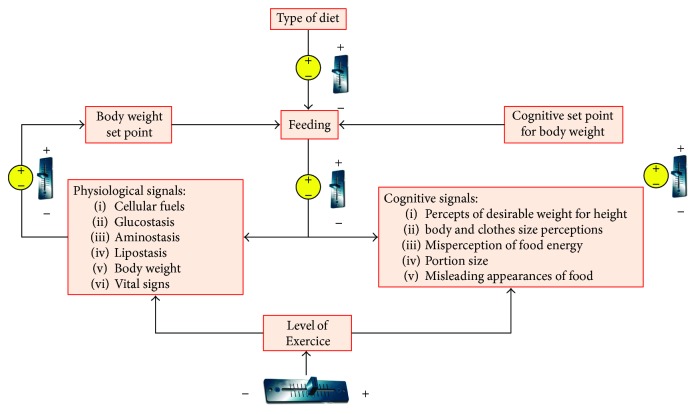
Combination of cognitive and physiological aspects to the better control of obesity.

Finally, we should take into account that the best way to combat obesity is to address the problem from two different points of view. In this delicate process, we must distinguish two aspects of training and teaching. On the one hand, the body weight set point considers all those physiological parameters that should be periodically measured to monitor the health status of a person [81, 82]. However, the cognitive and psychological aspects of this process also play an important role [83]. In this case, the cognitive set point evaluates the perception of the person with respect to their improvements. Both physical and cognitive aspects must be properly controlled to establish the type of diet, the amount of food that can be ingested, and the level of exercise that each person can make due to their physical condition.  Figure 11 shows the combination of physiological and cognitive parameters and how they can affect the course of a patient to achieve a healthy physical and mental condition. As we can see, it is a feedback system that adapts the levels of exercise and the diet requirement to the physiological values measured on the patient. However, both the level of exercise and the type of diet should be, in many cases, a little flexible, because the mood sometimes plays a very important role to continue with a diet. From the combination of the physiological and cognitive parameters and their evolution over time, it is established to set points to feed the system, readapt, and calculate the new parameters to successfully continue with the process of losing weight. The psychological perception of the situation and the patient's perception of his/her good evolution help him/her to continue with the diet or treatment.

It is obvious to think that to develop a good system able to monitor and control obesity, the system must combine both parts, that is, a good sensor network to control the parameters involved in the obesity measurements and an efficient algorithm capable of evaluating the data collected and take the most appropriate decisions to reduce overweight and obesity in people.

## 6. Conclusion

Many healthcare professionals predict that the prevalence of obesity would rapidly increase in the coming decade all over the world due to the lifestyle dependence on obesity-related factors such sedentary jobs, technology advancements, and unhealthy foods. The advancement in smartphone devices, technologies, and wireless sensor networks revolutionize healthcare domain by providing intelligent and low-cost m-health and WBAN systems that will facilitate communication with patients to acquire better services in the optimal time before the serious danger of diseases threatens their lives.

This paper attempts to study all about obesity including the related health problems due to obesity and overweight and methods used to determine the body fat percentage and its distribution.

We have also analyzed and discussed the current health-oriented WBANs and mobile applications specially designed to monitor people activity and eating habits. The current smartphone sensing systems have been developed to sound the obesity alarm by monitoring and delivering indication factors including daily physical activity level, sedentary time estimation, and food intake history reports anytime, as needed, and anywhere.

Finally, we have explained how a smart m-health system for obesity control should be designed to be efficient in the goal of obesity and overweight control. We agree that the best way of designing a system able to monitor and control obesity is combining both the physical measurements and cognitive positive feedback to motivate patients and demonstrate their evolution in the process of losing weight, re-educate their eating habits, and seek a healthier lifestyle.

As future features to be integrated into smart m-health system, we should consider the possibility of creating collaborative wireless networks to monitor several individuals and combining the data from all nodes to take more efficient decisions [84]. Finally, it is important to consider the secured aspects for protecting the sensitive data the network transmits [85]. With all these considerations and the huge evolution of wearable sensors and smartphones, we are sure we will be able to create efficient systems for combating obesity and overweight.

## Figures and Tables

**Table 1:** BMI classification.

Classification		BMI
Underweight		<18.5 kg/m^2^
Normal weight		18.5 kg/m^2^–24.9 kg/m^2^
Overweight		25 kg/m^2^–29.9 kg/m^2^
Obesity (class 1)		30 kg/m^2^–34.9 kg/m^2^
Obesity (class 2)		35 kg/m^2^–39.9 kg/m^2^
Extreme obesity (class 3)		>40 kg/m^2^

**Table 2:** Comparison of anthropometric measurement methods.

Measurement parameters	Method	Technology	Method-based technology advantages	Method disadvantages	Method advantages
Height and weight	BMI	Digital scale and tape measure	Inexpensive, require minimal training to use, virtually maintenance-free, and repeat values can be obtained with good precision	Not accurate method for assessing body fatness for the individual especially for children	Easy, fast, costless, and noninvasive measurements

Waist circumference	Waist and hip circumferences	Waist and hip tapes	—	—	Costless, noninvasive

Subcutaneous fat layer	Skinfold thickness	Skinfold mechanical calipers	—	Operator dependent and requires well trained person	Costless (10 US$/pair)
	Ultrasound	Scanning devices	Most accurate instruments	Provide the automation of analysis and reduce the operator-dependent errors	Only for clinical use
	Infrared interactance				
	Photon backscatter				

**Table 3:** Body composition method comparison.

Method	Underlying principle	Reliability (+ to +++)	Availability and cost	Advantages/disadvantages
*Anthropometric methods*				
BMI	Weight/height^2^	+ Inaccurate	The most used metric of obesity Costless	Cheap and it does not require special equipment
Skinfold thickness	Thickness of the skin at various body areas	++ Accurate but the operator needs specific training	Costless	Costless
Waist circumference and waist-to-hip ratio	Circumferential measurements of the abdomen and the hip	++	Costless Superior to BMI for prognostic purposes	Cheap and it does not require special equipment
Body adiposity index	An index based on the hip circumference and height measurements	++	Costless	Cheap and it does not require special equipment

*Machine-based methods*				
Underwater weighing	Body density	+++	For clinical research only, expensive	Cheap
Air displacement plethysmography (ADP)	Body density.	+++ But not practical and required specialized person	For clinical research only, expensive	It is highly expensive and demands special apparatus and the use of compression underwear
Dual-energy X-ray absorption	The intensity of the X-ray is associated with the density, thickness and chemical composition of the crossed object	++ Frequently used as a gold standard	Used mainly in clinical research, expensive	It requires taking an appointment with a medical professional in a clinic
Computed tomography (CT)	Transmitted intensity of X-rays	++ Mainly used as a gold standard	Used for clinical research only, highly expensive	Used for clinical research only, highly expensive
MRI	The relaxation time for protons in fat is much shorter than that for protons in water	+++ Mainly used as a gold standard	Used for clinical research only, very costly	Used for clinical research only, highly expensive
BIA	Based on the fact that fat is a relatively nonconductive tissue Impedance analysis	+ to ++	Instruments of various cost and reliability Multifrequency instruments Quiet and well validated	It does not need high technical skills to perform the impedance measurement
Near-infrared interactance	Optical densities are linearly and inversely related to percentage body fat	+	Inexpensive but insufficiently accurate	Its results in percent fatpredictability are slightly better than BMI

**Table 4:** Schema of food intake measuring methods.

Method	Measured parameter	Observations
Monitoring of swallowing	(1) Monitoring of sounds	Microphone placed on laryngopharynx/mastoid bone
	(2) Monitoring of motion of the larynx	Using accelerometers or magnetometers
	(3) Monitoring of changes in electric impedance across the neck at larynx level	Using electroglottograph (EGG)

Monitoring of chewing	(1) Electrical activity of jaw muscles	Using electromyography (EMG)
	(2) Sounds generated during the movements	Using a microphone placed on laryngopharynx/mastoid bone
	(3) Changes of jaw shape	Strain gauges/piezoelectric film

Monitoring of hand gestures	Monitoring hand-to-mouth gesture	Using gyroscope

Monitoring of gastric activity and physiological response to food intake	Using on-body and in-body sensors	Using on-body and in-body sensors

Monitoring of quantity and type of food	Quantity and type of food	Image processing methods

Monitoring of chemical food composition	Chemical composition of food	Spectroscopy

**Table 5:** Comparison of health-oriented WBAN solutions for obesity control.

No.	Paper	Fining	Number of sensor(s)	Communication technology	Sensor node	Year
1	[62]	Reducing sedentary behavior (lying down, sitting, and standing) and promoting physical activity in overweight Hispanic adolescents	Three types of sensors ACC, ECG, and OX and GPS	3G, EDGE, and Wi-Fi for three transmission phases	Nokia N95 mobile	2012
2	[63]	Monitore body motion, calculate calories burned, and provide intelligent suggestions	ACC	IEEE 802.15.4 standard for communication	iMote2	2014
3	[64]	Monitor user activity and health status	ACC	ZigBee	Telos	2005
4	[86]	Monitor user activity and heart rate	ACC and ECG	—	Tmote sky	2006
5	[66]	Able to epidemic source tracing	Mobile phone sensors	Bluetooth	—	2013
6	[67]	Encourage self-monitoring of daily dietary intake, PA, calories, and fat grams consumed	ACC, thermometer	Bluetooth	Mobile (Motorola)	2012
7	[65]	Vital signs monitoring	Applicable for any off-the-shelf sensors	ZigBee (IEEE 802.15.4) or Bluetooth (IEEE 802.15.1)	Tmote sky	2005
8	[68]	Health parameter control and disease risk assessment	It is a platform for integrating and combining signals from sensors	—	Smartphone	2013

**Table 6:** Summary of health-oriented mobile apps using MSP.

No.	Study	Fining	Number of sensor(s)	Sensor node	Year
1	[73]	Encourage regular physical activity	MSP	Mobile	2009
3	[74]	Sedentary behavior	MSP	WinMobile	2014
4	[75]	Monitor real-time caloric balance and PA	MSP	—	2007
5	[76]	Motivate users to lose weight, increase their physical activity, and gain balanced nutritional state	MSP	Smartphone	2011
6	[77]	Motivate walking alternatives to ordinary routes	Two accelerometers, GPS	Smartphone	2010
7	[78]	Measure the daily activities and to increase the user awareness	Sensor-enabled in smartphones	Smartphone	2009
8	[79]	Provide an accurate amount of daily food and nutrient intake.	MSP (camera)	Smartphone	2010
9	[80]	Educate the healthy eating habits	Camera	Smartphone	2010

## References

[1] School of Public Health (Harvard T.H. Chan) Obesity causes. https://www.hsph.harvard.edu/obesity-prevention-source/obesity-causes/.

[2] Kerry C. What is a healthy body fat percentage?. https://bodyfatloss.com/what-is-a-healthy-body-fat-percentage/.

[3] World Health Organization Noncommunicable diseases. http://www.who.int/mediacentre/factsheets/fs355/en/.

[4] Sazonov E., Neuman M. R. (2014). *Wearable Sensors: Fundamentals, Implementation and Applications*.

[5] Lee J. M., Kim Y., Welk G. J. (2014). Validity of consumer-based physical activity monitors. *Medicine & Science in Sports & Exercise*.

[6] World Health Organization Obesity data and statistics. http://www.euro.who.int/en/health-topics/noncommunicable-diseases/obesity/data-and-statistics.

[7] World Health Organization Obesity. http://www.who.int/topics/obesity/en/.

[8] Courtemanche C. J., Pinkston J. C., Ruhm C. J., Wehby G. L. (2016). Can changing economic factors explain the rise in obesity?. *Southern Economic Journal*.

[9] GDP per capita (current US$) In The world Bank web site. https://data.worldbank.org/indicator/NY.GDP.PCAP.CD.

[10] Grundy S. M. (2004). Obesity, metabolic syndrome, and cardiovascular disease. *The Journal of Clinical Endocrinology & Metabolism*.

[11] Jovanov E., Sazonov E., Poon C. Sensors and systems for obesity care and research.

[12] Liu J., Johns E., Atallah L. An intelligent food-intake monitoring system using wearable sensors.

[13] Rubenstein A. H. (2005). Obesity: a modern epdimic. *Transactions of the American Clinical and Climatological Association*.

[14] Douketis J. D., Macie C., Thabane L., Williamson D. F. (2005). Systematic review of long-term weight loss studies in obese adults: clinical significance and applicability to clinical practice. *International Journal of Obesity*.

[15] Calle E. E., Thun M. J. (2004). Obesity and cancer. *Oncogene*.

[16] Govindarajan R., Madan S., Shett A. Hadoop based obesity monitoring system. http://www.sjsu.edu/people/rakesh.ranjan/courses/cmpe272/s2/Team-Watson-Hadoop-based-Obesity-Monitoring-System.pdf.

[17] Burkhauser R. V., Cawley J. (2008). Beyond BMI: the value of more accurate measures of fatness and obesity in social science research. *Journal of Health Economics*.

[18] Freedman D. S., Wang J., Maynard L. M. (2005). Relation of BMI to fat and fat-free mass among children and adolescents. *International Journal of Obesity*.

[19] Leproult R., Van Cauter E. (2010). Role of sleep and sleep loss in hormonal release and metabolism. *Endocrine Development*.

[20] Wu Y., Zhai L., Zhang D. (2014). Sleep duration and obesity among adults: a meta-analysis of prospective studies. *Sleep Medicine*.

[21] Qidwai W., Ishaque S., Shah S., Rahim M. (2010). Adolescent lifestyle and behaviour: a survey from a developing country. *PLoS One*.

[22] Westerlund L., Ray C., Roos E. (2009). Associations between sleeping habits and food consumption patterns among 10-11-year-old children in Finland. *British Journal of Nutrition*.

[23] Leon B. G. C., Jensen M. D., Hartman J. J., Jensen T. B. (2016). Self-measured vs professionally measured waist circumference. *The Annals of Family Medicine*.

[24] Ellis K. J. (2001). Innovative non-or minimally-invasive technologies for monitoring health and nutritional status in mothers and young children. *Journal of Nutrition*.

[25] Barrios P., Martin-Biggers J., Quick V., Byrd-Bredbenner C. (2016). Reliability and criterion validity of self-measured waist, hip, and neck circumferences. *BMC Medical Research Methodology*.

[26] Zoccali C., Torino C., Tripepiand G., Mallamaci F. (2012). Assessment of obesity in chronic kidney disease: what is the best measure?. *Current Opinion in Nephrology and Hypertension*.

[27] Lobman T. G., Houtkooper L., Going S. B. (1997). Body fat measurement goes high-tech. *ACSM's Health & Fitness Journal*.

[28] Kullberg J., Brandberg J., Angelhed J. E. (2009). Whole-body adipose tissue analysis: comparison of MRI, CT and dual energy X-ray absorptiometry. *The British Journal of Radiology*.

[29] Kyle U. G., Bosaeus I., De Lorenzo A. D. (2004). Bioelectrical impedance analysis-part II: utilization in clinical practice. *Clinical Nutrition*.

[30] Karabulut M. (2004). *Validation and comparison of two ankle-mounted and two waist-mounted electronic pedometers, [Masters’ Thesis]*.

[31] Katzmarzyk P. T. (2010). Physical activity, sedentary behavior, and health: paradigm paralysis or paradigm shift?. *Diabetes*.

[32] Marshall S. J., Ramirez E. (2011). Reducing sedentary behavior: a new paradigm in physical activity promotion. *American Journal of Lifestyle Medicine*.

[33] Bonomi A. G., Westerterp K. R. (2012). Advances in physical activity monitoring and lifestyle interventions in obesity: a review. *International Journal of Obesity*.

[34] Giannini A. (2013). *Comparison of the Fitbit Zip to the actiCal accelerometer in children and adults, in biomechanics and movement science, [Doctoral Thesis]*.

[35] Vyas N., Farringdon J., Andre D., Stivoric J. I. (2012). Machine learning and sensor fusion for estimating continuous energy expenditure. *AI Magazine*.

[36] Yang C. C., Hsu Y. L. (2010). A review of accelerometry-based wearable motion detectors for physical activity monitoring. *Sensors*.

[37] Ananthanarayan S., Lapinski N., Siek K., Eisenberg M. Towards the crafting of personal health technologies.

[38] Ferguson T., Rowlands A. V., Olds T., Maher C. (2015). The validity of consumer-level, activity monitors in healthy adults worn in free-living conditions: a cross-sectional study. *Journal of Behavioral Nutrition and Physical Activity*.

[39] Rachele J. N., McPhail S. M., Washington T. L., Cuddihy T. F. (2012). Practical physical activity measurement in youth: a review of contemporary approaches. *World Journal of Pediatrics*.

[40] Mackinlay M. (2013). Phases of accuracy diagnosis: (in) visibility of system status in the Fitbit. *Intersect: the Stanford journal of Science, Technology and Society*.

[41] Pierce A. (2011). Digital technologies jump start telemedicine. *Tech Directions*.

[42] Withings, Pulse Ox Track Improve In Withings Inspire Health. http://www.withings.com/eu/withings-pulse.html.

[43] Kaewkannate K., Kim S. A. (2016). Comparison of wearable fitness devices. *BMC Public Health*.

[44] Bonomi A. G., Plasqui G., Goris A. H., Westerterp K. R. (2009). Improving assessment of daily energy expenditure by identifying types of physical activity with a single accelerometer. *Journal of Applied Physiology*.

[45] Bonomi A. G., Plasqui G., Goris A. H., Westerterp K. R. (2010). Estimation of free-living energy expenditure using a novel activity monitor designed to minimize obtrusiveness. *Obesity*.

[46] Gordon B. Jawbone UP review. http://fitness-trackers-review.toptenreviews.com/jawbone-up-review.html.

[47] Bonilla C., Brauer P., Royall D., Keller H., Hanning R. M., DiCenso A. (2015). Use of electronic dietary assessment tools in primary care: an interdisciplinary perspective. *BMC Medical Informatics and Decision Making*.

[48] Vereecken C. A., Covents M., Sichert-Hellert W. (2008). Development and evaluation of a self-administered computerized 24-h dietary recall method for adolescents in Europe. *International Journal of Obesity*.

[49] Steyn N. P., Jaffer N., Nel J. (2016). Dietary intake of the urban black population of cape town: the Cardiovascular Risk in Black South Africans (CRIBSA) study. *Nutrients*.

[50] Sazonov E., Schuckers S., Lopez-Meyer P. (2008). Non-invasive monitoring of chewing and swallowing for objective quantification of ingestive behavior. *Physiological Measurement*.

[51] Makinwa K. A. A. (2010). Smart temperature sensors in standard CMOS. *Procedia Engineering*.

[52] Sazonov E. S., Schuckers S. A., Lopez-Meyer P. (2009). Toward objective monitoring of ingestive behavior in free-living population. *Obesity*.

[53] Fontana J. M., Farooq M., Sazonov E. Automatic ingestion monitor: a novel wearable device for monitoring of ingestive behavior.

[54] Dong Y., Hoover A., Scisco J., Muth E. A. (2012). New method for measuring meal intake in humans via automated wrist motion tracking. *Applied Psychophysiology and Biofeedback*.

[55] Päßler S., Wolff M., Fischer W. J. (2012). Food intake monitoring: an acoustical approach to automated food intake activity detection and classification of consumed food. *Physiological Measurement*.

[56] Amft O. A wearable earpad sensor for chewing monitoring.

[57] Lopez-Meyer P., Makeyev O., Schuckers S., Melanson E. L., Neuman M. R., Sazonov E. (2010). Detection of food intake from swallowing sequences by supervised and unsupervised methods. *Annals of Biomedical Engineering*.

[58] Sazonov E. S., Makeyev O., Schuckers S., Lopez-Meyer P., Melanson E. L., Neuman M. R. Automatic detection of swallowing events by acoustical means for applications of monitoring of ingestive behavior.

[59] Amft O., Tröster G. On-body sensing solutions for automatic dietary monitoring.

[60] Fontana J. M., Sazonov E. S. A robust classification scheme for detection of food intake through non-invasive monitoring of chewing.

[61] Latré B., Braem B., Moerman I., Blondia C., Demeester P. A. (2011). Survey on wireless body area networks. *Wireless Networks*.

[62] Lee S., Annavaram M. Wireless body area networks: where does energy go?.

[63] Alrajeh N. A., Lloret J., Canovas A. (2014). A framework for obesity control using a wireless body sensor network. *International Journal of Distributed Sensor Networks*.

[64] Jovanov E., Milenkovic A., Otto C. A WBAN system for ambulatory monitoring of physical activity and health status: applications and challenges.

[65] Otto C., Milenkovic A., Sanders C., Jovanov E. (2006). System architecture of a wireless body area sensor network for ubiquitous health monitoring. *Journal of Mobile Multimedia*.

[66] Zhang Z., Wang H., Lin X., Fang H., Xuan D. Effective epidemic control and source tracing through mobile social sensing over WBANs.

[67] Pellegrini C. A., Duncan J. M., Moller A. C. (2012). A smartphone-supported weight loss program: design of the ENGAGED randomized controlled trial. *BMC Public Health*.

[68] Kartsakli E., Lalos A. S., Antonopoulos A. (2014). A survey on M2M systems for mHealth: a wireless communications perspective. *Sensors*.

[69] Ibarra E., Antonopoulos A., Kartsakli E., Rodrigues J. J., Verikoukis C. QoS-aware energy management in body sensor nodes powered by human energy harvesting.

[70] Otal B., Alonso L., Verikoukis C. Novel QoS scheduling and energy-saving MAC protocol for body sensor networks optimization.

[71] Metola E., Bernardos A. M., Casar J. R. A multi-device framework to increase self-awareness for health and disease prevention.

[72] Parra L., Sendra S., Jiménez J. M., Lloret J. (2016). Using multimedia sensors embedded in smartphones for ambient assisted living and e-health. *Multimedia Tools and Applications*.

[73] Klasnja P. V., Consolvo S., McDonald D. W., Landay J. A., Pratt W. Using mobile & personal sensing technologies to support health behavior change in everyday life.

[74] Bond D. S., Thomas J. G., Raynor H. A. (2014). B-MOBILE - a smartphone-based intervention to reduce sedentary time in overweight/obese individuals: a within-subjects experimental trial. *PLos One*.

[75] Tsai C. C., Lee G., Raab F. (2007). Usability and feasibility of PmEB: a mobile phone application for monitoring real time caloric balance. *Mobile Networks and Applications*.

[76] Lopes I. M., Silva B. M., Rodrigues J. J., Lloret J., Proença M. L. A mobile health monitoring solution for weight control.

[77] Brehmer M., El-Zohairy M., Jih-Shiang Chang G., Himmetoglu H. G. Walkabout: a persuasive system to motivate people to walk and facilitate social walks planning.

[78] Khalil A., Glal S. StepUp: a step counter mobile application to promote healthy lifestyle.

[79] Zhu F., Bosch M., Woo I. The use of mobile devices in aiding dietary assessment and evaluation.

[80] Pollak J., Gay G., Byrne S., Wagner E., Retelny D., Humphreys L. It’s time to eat! Using mobile games to promote healthy eating.

[81] McDonald L. Bodyweight regulation: leptin part 1. http://www.bodyrecomposition.com/fat-loss/the-hormones-of-bodyweight-regulation-leptin-part-1.html/.

[82] McDonald L. Set points settling points and bodyweight regulation part 2. http://www.bodyrecomposition.com/fat-loss/set-points-settling-points-and-bodyweight-regulation-part-2.html/.

[83] Linehan M. (1993). *Cognitive-Behavioral Treatment of Borderline Personality Disorder*.

[84] Lloret J., Garcia M., Catala A., Rodrigues J. J. (2016). A group-based wireless body sensors network using energy harvesting for soccer team monitoring. *International Journal of Sensor Networks*.

[85] Lloret J., Sendra S., Jimenez J. M., Parra L. Providing security and fault tolerance in P2P connections between clouds for mHealth services. *Peer-to-Peer Networking and Applications*.

[86] Otto C. A., Jovanov E., Milenkovic A. A WBAN-based system for health monitoring at home.

